# A grand unified theory for the unification of physics, life, information and cognition (mind)

**DOI:** 10.1098/rsta.2022.0277

**Published:** 2023-08-07

**Authors:** Rod Swenson

**Affiliations:** Center for the Ecological Study of Perception and Action, University of Connecticut, Storrs, CT, USA

**Keywords:** maximum entropy production, evolution, fourth law of thermodynamics, self-organization, information, 4E cognition

## Abstract

The modern scientific world view was built on the incommensurability between cognition (mind) and physics (matter) and later life and physics (the autonomy of biology). Fuelled by Boltzmann's view of the second law of thermodynamics as a ‘law of disorder’, the idea of ‘two opposing rivers’, the river of physics ‘flowing down’ to disorder and the river of life and mind ‘flowing up’ to higher states of order became a cornerstone of contemporary thinking. The deleterious result of this paradigmatic separation of physics, life and mind has been to considerably incapacitate each by bracketing many of the deepest problems of science, including the very nature of life itself and its cognitive capabilities, outside the theoretical reach of contemporary science. An expanded view of physics, notably the addition of the fourth law of thermodynamics (LMEP), or the law of maximum entropy production, coupled with first law time-translation symmetry, and the self-referencing circularity of the relational ontology of autocatakinetic systems, provides the basis for a grand unified theory unifying physics, life, information and cognition (mind). This dissolves the dysfunctional myth of the two rivers, and solves the previously insoluble problems at the foundations of modern science associated with it.

This article is part of the theme issue ‘Thermodynamics 2.0: Bridging the natural and social sciences (Part 1)’.

## 1. Introduction and background: reductive materialism and the roots of the modern scientific world view

Following the virulent attacks on Aristotelian teleology by Bacon^1^ and Descartes, the success of Newtonian mechanics paved the way for the modern scientific world view to be built almost entirely on efficient cause [1]. By contrast, the physics of Aristotle was inherently active, end-directed and multi-causal. The reductive materialism of this ‘purposeless particle’ world view effectively eliminated all goals, intentions and other end-directed behaviour from the physical world. Although Newton's physics eclipsed Descartes', it was the latter's metaphysics that set the foundation for the modern scientific world view, and where physics (matter) and psychology (mind) were defined at their modern origins by their mutual exclusivity (first postulate of incommensurability) [2–4]. ‘Mind’ (the perceiving mind, ‘thinking I’, or Cartesian ‘self’, *res cogitans*), the active, striving, end-directed part of the world was seen as unbounded in space and time and immune from physical law, while ‘matter’ (body, *res extensa*), the physical, ‘dead’ part of the world consisted of inert, reversible, quality-less particles defined exhaustively by extension in space and time and rigid deterministic laws. There are two immediate implicates of this.

The first is that a physics so reduced *by definition* needs extra-physical ordering or an outside ‘maker’ to order it. As the writings of Boyle and Newton indicate, this itself was used as an argument for the existence of God [1]. A world conceived as a giant, intricately well-ordered machine or clockwork, comprised ‘dead’ (reversible) particles requires a ‘watchmaker’ outside the physical world to order it. Humans, then, following Descartes, were dualistically situated with ‘mind’ miraculously outside the physical world and body within it manipulating the clock towards divine ends.

The second implicate is particularly notable because it fatally undermines the first. In particular, given the ontology the first postulate proclaims, *by definition* ‘mind’ (= all extra-physical makers) is *logically forbidden* from interacting with the physical world (matter) it is relied on to order. To act upon a world defined by extension an agent/agency must meet the physical world or part of it at a particular place in space and time to exert a force in some way upon it. But for a generalized Cartesian mind (an extra-physical thing) to do so would then mean becoming itself extensively defined and becoming that which it is not. Intuiting the first law of thermodynamics, Leibniz recognized that for one thing to interact with another there had to be something conserved over the interaction, and for an unextended lawless thing there is no such conservation. This general causal impossibility, the problem of Cartesian or ‘dualist interactionism’ is still found front and centre across the disciplines today [2].

Later, following Descartes, Kant, arguing that the active, end-directed striving of living things (their *telos*) could not be accounted for as part of the dead world of physics, promoted a second major dualism, the dualism between physics and life (the ‘second postulate of incommensurability’) [5]. This view, the ‘autonomy of biology’ from physics, has remained entrenched in the modern scientific world view right up to the present times. Mayr, a flag waver for this view, adopted the word ‘teleonomy’ to replace teleology and defined it as ‘systems operating on the basis of a program of coded information’ [6, p. 42], which is then explained by natural selection. The flaw in relying on natural selection in this way is discussed more fully below.

It was Boltzmann's reduction of the second law of thermodynamics to a ‘law of disorder’, however, because it purportedly came from physics itself, that really reified the postulates of incommensurability in the modern scientific world view. Previously the world was supposed to consist of ‘dead’ reversible particles requiring ordering, but now the physical world was not just passive but instead incessantly working to destroy order. With the planetary origin and evolution of life showing the progressive (going in a direction) production of increasingly more highly ordered states now apparently running contrary to the laws of physics the view of the so-called two opposing ‘rivers’, the river of life and mind ‘flowing up’, and the river of physics ‘flowing down’, became fully entrenched into the modern scientific world view [7].

## 2. The first and second laws of thermodynamics: the physical basis for persistence and change

By the middle of the nineteenth century, evidence from geology and palaeontology was undermining the immutable mechanical world view and the subject of evolution had become widespread [3,5]. At the same time a series of deceivingly simple experiments led to the discovery of the first law of thermodynamics, and shortly thereafter the discovery of the second law. The profound nature of these laws, very special laws distinct from all the other laws of physics, has been often missed or obscured in their cooption under the Cartesian paradigm. The laws, which physicalize persistence and change, or more deeply invariance and end-directedness *through that invariance*, showed the entailment of a *relational ontology* from first principles [7,8]. They are thus special laws of nature because, in a very literal sense without them, for example, the time-translation symmetry of the first law there could be no other laws at all. The second law expresses a symmetry too, but one that governs the first law as well. For this reason, many, e.g. Eddington [9], have called it the ‘supreme law of nature’.

### (a) The first law and the unity of all things

**Figure 1.**
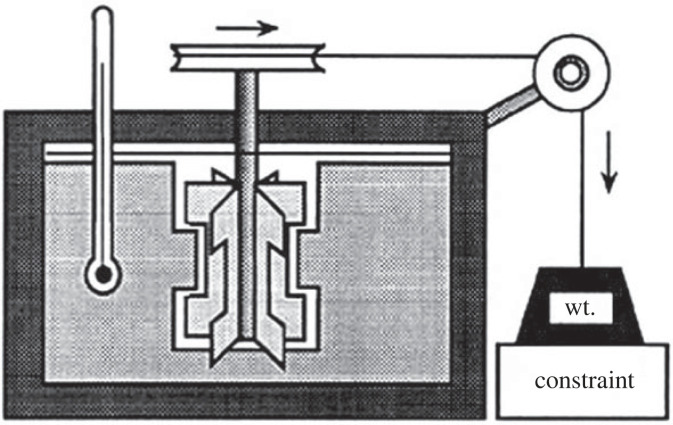
Joule's paddle wheel experiment. An elevated weight (a mechanical potential) attached to a paddle wheel sitting in an adiabatic (no heat flow in or out) container of water and held in place by a constraint. When the constraint is removed the weight falls, the paddle wheel turns elevating the temperature of the water demonstrating the equivalence of mechanical energy and heat while the total energy is conserved [4].

For the first law, the lynchpin was the discovery or observation and then demonstration of the equivalence of mechanical energy and heat [4]. Previously heat was considered a separate and conserved quantity (the ‘caloric’) and it was the falsifying of this view that opened the floodgates. Rubbing ice cubes together Davy realized *produced heat* which accelerated their melting. Mayer demonstrated the same thing by raising the temperature of a jar of water by shaking it. Mayer's attempt to publish his results was rejected and it was Joule with his more precise paddle wheel experiment that finally pushed it over the edge (figure 1). The discovery of the first law showed the unity of all natural processes, namely that all forms of energy are all interconvertible into each other while the total amount is always conserved (never created or destroyed). Not often noted, this vindicated Leibnitz’s argument against the separation of physics (matter) and psychology (mind) and the impossible problem of Cartesian interactionism [3].

### (b) The second law: physicalizing change ('the flow') as end-directedness comes nomologically into the world

Earlier working to understand the force that made steam engines go, Carnot [10] came away with a notable understanding, but one that left a hanging question concerning the first law. Carnot's insight was that it was the ‘fall’ of heat (from higher to lower temperature), he analogized, like the fall of water from higher to lower that drives a mill wheel, that produced the ‘motive’ force or the ‘availability’ as he called it, that drives a steam engine. But he also noted that in such transformations this availability is lost (water at the bottom of the mill wheel will not drive it anymore). Clausius [11] and Thomson (Lord Kelvin) [12] recognized explicitly that the take away from Carnot's results was that if there was a quantity conserved (the first law), then there had to be another that was not. Clausius coined the term ‘entropy’ to refer to the inverse of Carnot's availability (potential), and the second law, in its most general form thus says that *all* natural processes proceed so as to maximize the entropy (or equivalently to minimize the potential or availability). Put most simply, in all natural processes the entropy always increases. So for the universal statement given by Clausius, with the universe conceived as an isolated system (or for any isolated system within it), the ‘balance equation’ of the second law is thus

2.1 ΔS>0, or at equilibrium where ΔS=0.

The active macroscopic nature of the second law seemed like a death knell for the reversible ‘dead’ (inactive) mechanical world view giving scientific meaning to a physics based on end-directedness. ‘The universe,’ Clausius [11] wrote (in an often misquoted phrase^2^), ‘strives (*strebt*) to increase its entropy to a maximum’. While the first law is a law of invariance the second law says that for non-uniform distributions of this conserved quantity there is a symmetry unfulfilled, a broken symmetry (time-reversal symmetry) that the world acts spontaneously to fulfil.

### (c) The second law as a law of disorder: the mechanical world view gets life support with Boltzmann's statistical interpretation of the second law

#### (i) Boltzmann and the ‘infinite improbability of order’: the world as a gas in a box

The active macroscopic nature of the second law presented a direct challenge to the mechanical world view that Boltzmann tried to meet by reducing it to a law of probability following from the random collisions of mechanical particles. Following Maxwell, modelling gas molecules as colliding billiard balls in a box, Boltzmann noted that with each collision non-equilibrium velocity distributions (groups of molecules moving at the same speed and in the same direction) would become increasingly disordered leading to a final equilibrium state of macroscopic uniformity and microscopic disorder, the state of maximum entropy. The second law, Boltzmann argued could be reduced to the fact that in a world of mechanically colliding particles disordered states are the most probable. Because there are so many more possible disordered states than ordered ones, the system will almost always be found either in a state of maximum disorder, the macrostate with the greatest number of accessible microstates, such as a gas in a box at equilibrium, or moving towards it making transitions from disorder to order ‘infinitely improbable’ [13]. Molecules, wrote Boltzmann,

*moving at the same speed and in the same direction is the most improbable case conceivable…an infinitely improbable configuration of energy….*

Following Boltzmann's re-casting of the second law as nothing but a law of probability reduced to efficient cause and a ‘law of disorder’, the mechanical world view got a temporary reprieve from the macroscopic end-directedness recognized by Carnot, Clausius and Thomson. Correspondingly, the vision of the ‘two rivers’ became even more deeply entrenched. Fisher [14], one of the founders of Neo-Darwinism, opined ‘entropy changes lead to progressive disorganization…while evolutionary changes lead to progressively higher organization’, and Levins & Lewontin [15] proclaimed ‘organic evolution to be the negation of physical evolution’. Dennett [16], even more recently, in support of his computationalist or algorithmic (rule-based) view of cognition (mind) and life, described them as things that ‘defy’ the second law, and Friston [17] in support of a Bayesian information-theoretical account of living, cognitive systems (mind) characterized them as systems that ‘somehow manage to violate’ the second law.

## 3. The what, why and how of life? The shortcomings of Darwinism as the theory of evolution

Currently taken to be *the* theory of evolution, Darwinian (or Neo-Darwinian) theory has remained shackled by its commitment to an autonomy of biology position to this day. Evolution in neo-Darwinian terms is taken to be *explained* by natural selection and natural selection is explained by a situational logic [18]. That is, *if* certain conditions hold *then* natural selection will necessarily follow. These are (i) the ‘fecundity principle’, a biological extremum and the *sine qua non* of Darwinian theory expressing in Darwin's terms the ‘striving’ or ‘struggle’ of every living thing to fill or ‘seize on every unoccupied or less occupied space in the economy of nature’ [19]; (ii) finite resources (Malthusian closure); and (iii) heritable variation. From these three *necessarily* follows a ‘struggle for existence’ leading to adaptation and the ‘survival of the fittest’. But note, this puts the active striving of living things to fill out the economy of nature outside the explanatory framework of Darwinian theory [20]. Rather than explain it, natural selection depends on it to operate. The origin and nature of living things must simply be assumed *ad hoc* outside the theory. ‘Evolution’ for Darwinian theory thus begins with life and all its active properties as given rather than explained.

**Figure 2.**
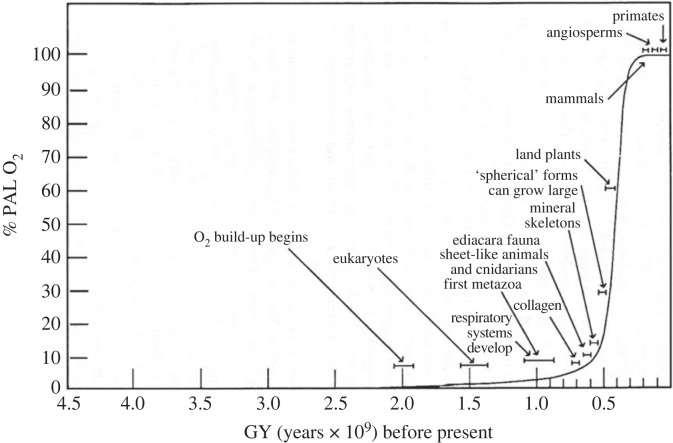
The opportunistic production of increasingly higher-ordered states as a function of increasing levels of atmospheric O_2_ (PAL = present atmospheric level) [21].

For Darwin life was ‘breathed in’ to dead matter by the Creator [19]. More recently, assuming a vast amount of time, e.g. 1–2 GY, thought to have elapsed before an ‘origin event’ took place, it was said to have occurred with an infinitely improbable accident ‘that only had to happen once’ [5]. But recent paleobiological discoveries have defeated this account showing life emerged not after some long time, but opportunistically almost immediately as soon as the Earth had cooled enough after its formation to keep the oceans from evaporating, and opportunistic ordering continued from there, e.g. multicellular eukaryotes as soon as there was enough oxygen (O_2_) in the atmosphere to support them, and larger forms as soon as O_2_ levels increased further (figure 2) [21].

**Figure 3.**
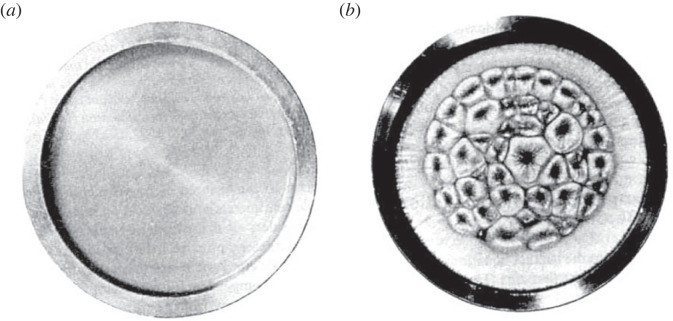
Two time slices from the Benard cell (BC) experiment show a viscous liquid held between a uniform heat source below (source) and cooler air above (sink) where the temperature difference between them produces a potential with a thermodynamic force ***F****.* When ***F*** is below a critical threshold the system (*a*) is in the disordered or ‘Boltzmann regime’, the flow of heat is due to the disordered collisions of molecules and the macrostate appears homogeneous. By contrast (*b*) as soon as ***F*** is increased above the threshold *macro is selected from micro* (ordered from disordered flow) and hundreds of millions of molecules begin moving collectively together [5].

Beyond the fact that Darwinism cannot explain the very nature of life, the active, end-directed striving to fill out the economy of nature, there is a cascade of related problems that follows from its bracketing off from physics and briefly noted here (for more detail see [3–5,8,20]). The first is the ‘problem of the population of one’ [5,21], the origin problem just discussed being a special case. The name comes from the instance of planetary evolution. During the latter half of the last century, it became clear that the Earth at the highest level has evolved as a single global entity [3,5,21], the immediate *prima facie* evidence for this being the creation and maintenance of planetary atmospheric O_2_ on which all higher ordered living forms depend, and put there by life itself (figure 3). Evolution with this had become a *coherent* planetary process with all other forms of life as component productions, and the fundamental unit of terrestrial evolution now had to be seen as the planetary system itself.

Yet for Darwinian theory, this is a major problem because from its view the planet cannot evolve or be considered a unit of evolution at all. Defining evolution as the product of natural selection acting on a competitive replicating or reproducing population of many it cannot recognize planetary evolution because there is no replicating or competing population of Earth systems on which natural selection can act [22,23]. The Earth evolves as a *population of one*. Both the origin of life and planetary evolution are special cases of the ‘problem of the population of one’; examples of the deeper problem of spontaneous ordering itself. All spontaneously ordered systems originate, as unpacked more fully below, in micro to macro transitions, as populations of one and Darwinian theory has no account of this at all. It has no theory of ordering *per se*.

There are other tributaries of this same problem that further flesh out the dysfunction of Darwinian theory as a result of bracketing itself from physics. Briefly (but see refs for detail, e.g. [3,4,20]) the first is the question of ordinality or the direction of evolution. The progressive nature of evolution is easy to see (e.g. figure 3), but orthodox Darwinian theory maintains that evolution does not have any kind of direction at all [24]. It is a time-symmetric theory with no observables within its theory that can be used to measure the direction of evolution or even recognize it. Because fitness is relativized to members of breeding populations, the fitness of different kinds of things are incommensurable with each other and cannot be compared, e.g. the fitness of a zebra cannot be compared with that of a mouse or an amoeba [25].

Finally, one cannot move on without noting the failure of Darwinian theory without a theory of ordering to address the whole of cultural evolution, that is human social evolution and artefact development, from hunter–gatherer tribes to the rise of nation states and the accelerating global ordering going on today [3,5]. The symmetry break that characterizes the increase in rate and scale of planetary ordering happening right now in the current Anthropocene does not even show up on Darwinian radar. Darwinism is defined as a change in gene frequencies following from natural selection, and this again puts cultural evolution (which is not defined by changes in gene frequencies, but happens at a much, much faster rate) outside the reach of Darwinian theory.

## 4. Turning the second law as a ‘law of disorder’ on its head: physical selection or why the world is in the order production business

### (a) The world is not a gas in a box and the Boltzmann reduction is readily falsified

There are any number of versions in theory forensics, deconstruction or theory selection of the common sense maxim that if everywhere you look the world goes contrary to your theory, then more likely your theory is wrong rather than the world. The utility of statistical methods in physics as a heuristic device aside, Boltzmann's attempt to reduce the second law to *nothing but* the statistical result of mechanically colliding particles is easily falsified by simple experiments. The second law, simply put, does not reduce this way.

Figure 3 shows two time slices from the now classic Benard cell (BC) experiment from our laboratory some decades ago [1,5,8,19]. A viscous liquid is held between a uniform heat source below (source) and the cooler air above (sink). The difference between the temperatures constitutes a potential with a thermodynamic force ***F*** the magnitude determined by the steepness of the gradient between them. When ***F*** is below a critical threshold the system (figure 3*a*) is in the linear disordered ‘Boltzmann regime’, and the flow of heat from source to sink is from the disordered collisions of molecules and the macroscopic state appears homogeneous. But (figure 3*b*) as soon as ***F*** is increased above the threshold *macro is selected from micro* and hundreds of millions of molecules begin moving together as a new highly ordered macrostate comes into being. According to Boltzmann's reduction of the second law such transitions are infinitely improbable (§2c(i)), but this claim, as is easy to see here, is entirely falsified by this simple experiment where *order arises not infinitely improbably but with a probability of one*, that is opportunistically every time and as soon as it gets the chance (every time ***F*** is above the critical threshold). There are a lot of generic takeaways from this simple experiment, but the bottom line here is that it entirely falsifies Boltzmann's reduction. The second law cannot be reduced to a law of disorder, or a stochastic collision function. The world is not a gas in a box [1,3,5,20,26].

### (b) Physical selection: macro from micro

#### (i) Identity *through* flow: the relational ontology of autocatakinetic systems

The BC is a member of the class of ‘autocatakinetic’ (ACK) systems, a more precisely defined term than ‘self-organizing system’ or ‘dissipative structure’ historically often used for the same kind of system. The latter, however, lacking clear definitions, are also used for very different systems that are not members of the class (see appendix A). We use the term ACK to make the important distinction. ACKs are flow structures, their identities constituted through flow, and defined as

a system that maintains its ‘self’ as an entity constituted by and empirically traceable to a set of nonlinear (circularly causal) relations (constitutive relations) through the dissipation or breakdown of environmental potentials (resources) in the continuous coordinated motion of its components [4,5,7,8].

From the definition, it is simple to see that all living systems from cells to ecosystems at whatever scale (including the planet itself) are ACK systems as are abiotic systems such as dust devils, tornadoes and experimental systems like the BC. What this definition explicitly does not include are machines, artefacts or systems that run exclusively on rules (e.g. algorithms) or rule execution. All these systems, ideal or not, are found to be component processes of ACKs but they are not ACKs themselves.

**Figure 4.**
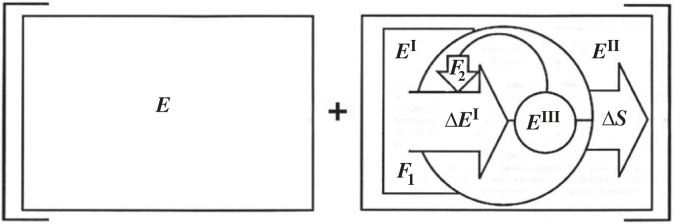
A canonical autocatakinetic (ACK) system. ***E*** (left side) is the conservation (energy, ‘world’) *out of and through* which an ACK arises. ***E^I^*** and ***E^II^*** indicate a source (an out of equilibrium region of ***E***) and sink (an equilibrium or closer to equilibrium region of ***E***) the gradient between them constituting a potential with a force ***F*_1_** the magnitude determined by the steepness. Δ***E^I^*** is the flow of energy at the input or drain on the potential which is transformed into entropy Δ***S*** at the output. ***E^III^*** is the internal (on board) potential carried in the constitutive relations of the system by virtue of its distance from equilibrium acting back with a force ***F*_2_** to amplify or maintain the input. (From auto-‘self’ + cata ‘down’ + kinetic ‘of the motion of material bodies and the forces and energy associated therewith’) [4,7].

Figure 4 shows a schematic of the ‘minimal ontology’ (minimal description) of a canonical ACK presented as a conjunction to underscore the irreducible *relational ontology* we are talking about with such a system. The constitutive relations that distinguish it as an entity, as a ‘self’, originate and are maintained *out of and through* the conservation (energy, ‘world’), ***E***.

The ‘selfishness’ of ACK systems, the ontological basis for using the term ‘self’ is *causally* manifested and located in the circular relations that constitute them. They are deviation-amplifying systems [27], or thermodynamically speaking, *self-amplifying sinks* that feedback (***F*_2_**) internally developed forces to amplify their own input pulling resources (potential) into their own production or progressive ordering [20].

#### (ii) Von Bertalanffy, Schrödinger and Prigogine

Von Bertalanffy [28], under the rubric of ‘open systems’, and Schrödinger [29] comparing living things to flames, helped to soften the physics versus biology problem by pointing out that as long as living systems produced enough entropy to compensate for their own internal entropy reduction (the order that defines them or potential they pull into themselves) so as to satisfy the balance equation of the second law (2.1) then the second law as classically stated would not be violated. Later Prigogine [30] made the same point under the term ‘dissipative structure’. The balance equation can then be separated into two terms. The first term is a measure of the change in entropy due to the ‘negentropy’, using Schrödinger's term, or the potential (energy gradients) on which such systems ‘feed’ and import into the system, and the second term is the entropy produced and dissipated into the environment by the system's ordering, and departure from equilibrium

4.1 $$\Delta S_{e}+\Delta S_{i}>0.$$

Living things and all non-living ACKs on this view are *permitted* to exist as long as they pay their ‘entropy debt’ [4,5]. This contributed to the recognition of living things as flow structures, but left the main problem still unsolved.

Permitted to exist, yes, but why should they? What explains their opportunistic origins, the urgency toward existence that brings them into being, motivates them while developing progressively further from equilibrium to begin with? The entire pattern of the origin and evolution of life and mind is characterized by this behaviour. What is the universality that explains, in effect, why the world is the order production business?

#### (iii) Order from disorder or macro from micro and the need for a physical selection principle

The first step towards the answer is to understand that spontaneous ordering (the production of ACK states), as a micro (disordered) to macro (ordered) transformation, *is a process of selection* [20,21]. By definition, it is the selection of some much smaller number of accessible microstates **M**_2_ (the new ordered macro state) from some initially much larger set **M**_1_. This can be equivalently understood as the spontaneous production of constraints on the previously disordered or less ordered microstates dramatically constraining their degrees of freedom in the production of the ordered flow. In short, *selection is entailed by autocatakinetics (spontaneous ordering)* [20], viz. *if* the latter has happened/is happening *then* a process of selection has/is taken(ing) place. But, clearly, the fact that this selection is not between replicating entities (biological or ‘natural’ selection), while at the same time observed in simple physical systems forces the insight that there must be an overriding physical selection principle to account for it.

#### (iv) Autocatakinetic closure and the expansion of space–time

**Figure 5.**
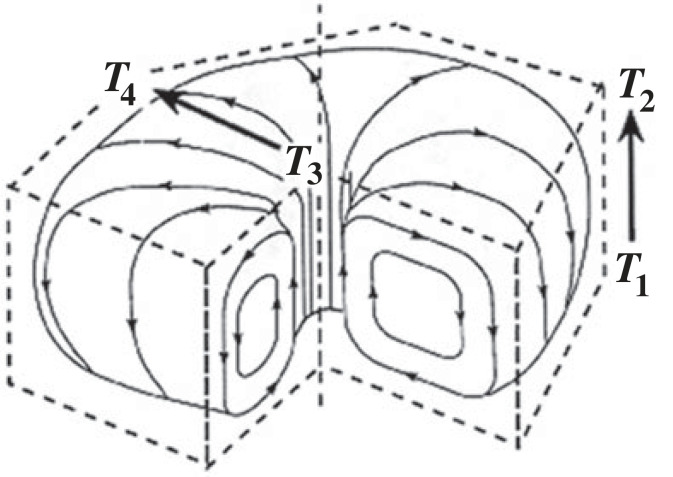
A generalized schematic of a BC. ***T*_1_** → ***T*_2_** is the original temperature gradient between the source (below) and sink (above) with a force ***F*_1_** (measured by the steepness of the temperature gradient) that motivates the flow. ***T*_3_** → ***T*_4_** shows the developed surface temperature gradient with a force ***F***_2_ that acts back to amplify the instantiation of the structure. With this spontaneous ordering the space–time dimensions of the system are increased by many orders of magnitude [20].

The second step towards the solution to the universal ordering problem is given by a more detailed look at what happens structurally with the individuation, or entification and progressive ordering of an ACK. The origin of such a system occurs following a symmetry-breaking event when *autocatakinetic closure* takes place and a new kind of causality, the self-amplifying circular closure of the constitutive relations that define the system as distinct literally comes into being (figure 5, and figure 4 for the generalized constitutive flow schematic) [3,5].

Figure 5 shows the macroscopic flow structure of a BC as hundreds of millions of previously randomly colliding molecules move together. ***T*_1 _→ *T*_2_** is the temperature gradient between source and sink that motivates the flow. Because density varies inversely with temperature there is also a density gradient from bottom to top giving groups of molecules (parcels) displaced upward by stochastic collisions (‘fluctuations’ = ‘deviants’ or ‘outliers’) an upward buoyant force. If the gradient is steep enough such that the parcel carries the heat *faster* towards the heat sink above than can be dissipated by its surrounds we see the beginning of macro being selected from micro as the parcel carries the heat to the surface sink. The upward flow of heat with the parcel at the same time increases the temperature of the upper surface directly above it creating a surface temperature gradient ***T*_3 _→ *T*_4_** which acts to further amplify the macroscopic upward flow of heat by pulling the hotter fluid to the cooler surroundings. Here it falls to the bottom where it becomes heated again to continue the constitutive circular relations of the cell and the autocatakinesis that brings it into being and defines it [20].

We are closer to the piece we are looking for, but to understand this more fully one needs to understand structurally or dimensionally what has occurred in this ordering process, and it is that the space–time dimensions of the system have increased by orders of magnitude. In the Boltzmann regime, the actual only measurable dimensions are the mean free path distances and relaxation times (the average times and distances between collisions) of the order of 10^−10^ cm and 10^−15^ s, or nanoscale, while in the ordered macro regime measurable dimensions or correlation between components increases to centimetres and seconds. If the micro mode were roughly scaled to human size, the macroscale would be many times greater than the circumference of the Earth developing and persisting of time scales greater than the 4.5 GY of planetary evolution. What does it mean?

#### (v) Final clue from the split balance equation of the second law

Revisiting the split balance equation of the second law (4.1) and the general point made by von Bertalanffy, Schrödinger and Prigogine, but in a whole new light, we immediately find something leading [31]. In particular, the significant point here is not that ordered flows are permitted to exist as long as they produce enough entropy to compensate for their internal entropy reduction. It is that with selection of macro from micro (spontaneous ordering) to occur *the rate of entropy production must always go up*. In fact, in more illuminating terms, the more order produced the greater the rate of entropy production is going to be.

**Figure 6.**
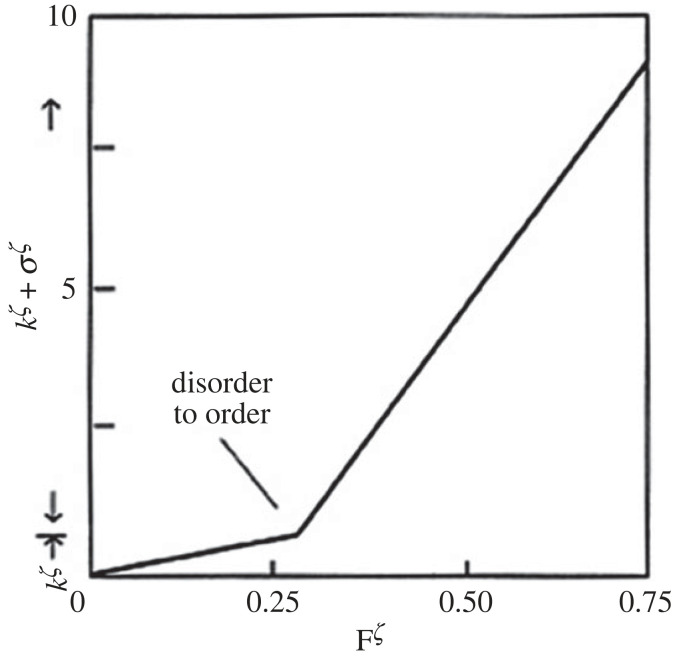
The discontinuous increase in the rate of heat transport during the disorder-to-order transition in a simple fluid experiment similar to that in figure 4 (heat flux plotted against the source/sink gradient) [4].

Figure 6 shows the dramatic increase in the rate of entropy production (as heat transport from source to sink) occurring in the transition from disorder to order in a simple experiment similar to the BC in figure 4 further highlighting what we saw in detail above during the production of ACK. It is path selection by rate, and with this the answer to the core question ‘why the world is in the order production business’ is virtually proclaiming itself [4].

#### (vi) The fourth law of thermodynamics (LMEP) or why the world is in the order production business

The previous sections take us directly to the physical selection principle that accounts for spontaneous ordering. It is the answer to the question that von Bertalanffy, Schrödinger and Prigogine never asked, and it is about *path selection* or rates. Specifically, ‘which paths out of available paths will a system take to get to equilibrium (maximize the entropy or minimize potentials)?’ The second law, of course, is mute on the subject. It only says that in all natural processes the entropy increases. The answer to this question, and the one that solves the entire question of physical selection, the ‘why’ of universal ordering, life and cognition is the law of maximum entropy production (LMEP) or the fourth law of thermodynamics [1,4,5,26,31–33]:

(the world) a system will select the path or assembly of paths out of available paths that minimizes the potential or maximizes the entropy at the fastest possible rate given the constraints

The fourth law says nothing directly about spontaneous ordering *per se*, but coupled with the balance equation of the second law the derivation of the universal ordering principle is easy to see, namely:

**IF** The 4th Law or LMEP (the world selects paths….fastest rate..)

**AND IF** ordered flow produces entropy at a faster rate than disordered flow (the balance equation of the 2nd Law)

**THEN** the world can be expected to select order from disorder whenever it gets the chance

And of course, this is just what happens. Like the first and second laws, LMEP meets the most rigorous standard possible in science, namely, stated in a way that subjects it to falsifiability with simple physical experiments, and like the first and second laws, the fourth law has never, in the three and half decades since it was formulated, been falsified (e.g. [26,31,32]). LMEP, which substantially expands thermodynamics is universal in scope applying near and far from equilibrium to all ranges and scales [26,31] (see appendix B).

## 5. The evolution of life, information and ‘mind’ (cognition)

LMEP when coupled with the relational, self-referencing and inherently self-amplifying circular causality of autocatakinesis (ACK + LMEP = ACKLMEP) dissolves the problem of the ‘two opposing rivers’ and gives us the universal (physical) basis for the nature of life to fill out the dimensions of space–time captured in the biological extremum or fecundity principle on which natural selection depends [5,20,34]. It gives us the agential or motivated activity missing in theories of cognition (mind) and its evolution including the most recent 4E post-computationalist theories [27,35] whose Cartesian dualist ancestry continues to bleed through. But while LMEP gives us ACK systems, and ACKLMEP most significantly thus gives us the urgency towards existence, the filling out of space–time and the generics of spontaneous ordering, it does not give us the special cases of life and cognition.

### (a) What particularly and why particularly is life?

All living/cognitive systems are ACK systems, but not all ACK systems are living or cognizing. What is it that distinguishes living from non-living systems (i) observing them from outside; or (ii) observing them inside? The short answer to (i) is ‘intentionality’, a term which needs immediate decontamination from the idea of ‘ends-in-mind’ (confined to human mental states a very lately evolved case). Expanding on Brentano [36], we define intentionality as end-directed behaviour prospectively controlled or determined by semantic content or meaning [37,38].

We do not invoke intentionality, or meaning, to explain the flow of a river down a slope or heat down a gradient, or the ‘selfish’ behaviour of abiotic ACKs pulling resources into their own ordering because these processes are determined by local force fields and potentials. By contrast, when a bacterium swims *up* a gradient its behaviour is seen to go in a direction that is oftentimes different, if not directly opposite to that determined by local potentials. Unlike rivers, or ACKs like the BC which are captives of their local gradients or potentials, e.g. remove the heat source of a BC and it ‘dies’, living systems behave arbitrarily with respect to their local potentials [5]. If a food supply is cut off say from a bacterium it may go dormant until a new potential arises, or its activity may actually increase as it seeks to find new discontinuously located potentials to feed on.

This, the intentionality of living things, is life's central distinguishing feature. Living systems are epistemic (cognitive) systems that constitute their ACK over times and distances that are arbitrary with respect to local potentials using instead their ‘on board’ potential (***E^III^*** in figure 1) and *information* (in the semantic or meaningful sense)^3^ to seek out and access non-local potentials and access otherwise inaccessible dimensions of space–time [5]. The dramatic increase to otherwise inaccessible dissipative dimensions afforded by the origin and progressive ordering of life and its cognitive functioning answers the ‘why’ question in the specific case. Rather than being incommensurable with the physical world as Cartesian metaphysics has it, cognition, or intentionality, and perception, the growing epistemic dimension of the world, or mind in nature, is seen instead as a direct manifestation of it [27,39].

### (b) The first law, time-translation symmetry and the physical basis for information

There is still an important missing piece. Life is distinguished by end-directedness determined by meaning or information, but as much as ACKLMEP and its active, ‘selfish’ relational ontology gives us, the ontological ground for the information itself still needs to be given. Specifically, where does meaning come from in a physical world of ‘meaningless’ particles? Cartesianism and heirs give us some version of the Cartesian circle with ‘mind’, having no possible relational connection to an outside world, simply perceiving itself. Information is something created or constructed in ‘minds’ not something otherwise existing in the world. Such a view, the idea that perception is essentially a creative process of mental operations or computations inside ‘minds’ or brains is still the dominant view today. CTM (the computational theory of mind) is a widely held form of this. ‘Perceiving as predicting’, a current leading trend in computational neuroscience, takes perception to be the probabilistic computation of models created in the brain. Adherents of this view, who as already noted assert that living things violate the second law [17], proclaim a ‘deep unity between perception and imagination’ [40]. Divorced from thermodynamics all of these views have the identical problem accounting for the agency, active striving or motivated activity that account for life and its cognitive properties, and none of them including 4E versions of cognition has an account other than being imaginatively created as to how meaning comes into the world, or if it did how an epistemic or cognitive agent could connect with it.^4^

Finally, beyond these problems, cognition ‘in brains’ models, have the further problem that cognitive functioning as noted being coextensive with life begins long before there were human brains or brains at all. Perception is clearly something much, much deeper and more fundamental than this. It is an entailment of intentionality, the definitive means by which a living thing establishes and maintains its ACKs. But, so where *is* the information?

The key was given by Gibson [41] and his ecological approach to perception. Perception, as discussed, is exactly the means by which a living thing coordinates its behaviour with respect to non-local potentials, but is not a mental construct at all but rather direct and law based. Constructions, imaginings and models are things humans and other forms of life may use to various degrees, but from this view, this is distinct from perception. Gibson's central insight was that living things are surrounded by ambient energy flows (e.g. optical, mechanical, chemical) for which the mean energy content is extremely low relative to the on-board potentials they use to power their intentional acts [5]. What he recognized was that by virtue of the invariant properties of such flows they lawfully carry information about their distal sources for the prospective control of an epistemic agent's intentional ends (e.g. bacterial chemotaxis and phototaxis). Perception is of that information, and as such is law-based, direct and done without internal representations, computations or inferences.

Now Gibson made no explicit connection to thermodynamics at all but what he did have was a remarkable and ground-breaking theory of perception and the law-based nature of information. This is simply because what Gibson had identified with his notion of information, without explicitly stating it, was the time-translation symmetry of the first law [4,27]. What this remarkable insight does in simple terms is to allow us to cross the final bridge to show the physical basis, coupled with the fourth law and ACK, for information, cognition and intelligence.

### (c) Information, replicative ordering and rate-independent constraints

The second part of the question in §5a, that is, what is life if you looked at it from the inside remains unanswered. Living systems are *replicative systems* (cf. replicat*ing* systems), namely ACK systems with internally replicating components [4,5]. Beyond being constituted by the flux of their components, they are *component production systems*. Behind the signature ability of living things to behave arbitrarily with respect to local potentials is the ability to maintain an arbitrary function in the component production process, and this is accomplished with a requisite set of rate-independent constraints (RICs). In particular, a set of internal constraints that are discrete, sequential and rate-independent relative to the rest of the ACK cycle. The order of the sequences, like the words on this page (a higher-order instantiation of this), or the sequence of base pairs in a DNA string, for example, is *thermodynamically arbitrary* relative to the rate at which they are ‘written’ and ‘read’ (viz., the amount of ATP used to replicate a string of DNA of the same length, is the same regardless of the particular sequence). Looking inside of an ACK system, *if* it has these features, that is component replication with a set of RICs, *then* the system is a living/cognizing system.

Understanding the function of RICs, it is worth noting, with genes and algorithms *sensu stricto* as notable examples, turns completely upside down all the various forms of ‘magic maker’ reductionism that wants to smuggle all the active agency and intentional dynamics of living things or ‘mind’ into the world *ac hoc* with ‘selfish replicators’ (e.g. [22]), or algorithms [16,20,34]. Rather than being active, striving or imbued somehow with agency, the entire function of RICs (including DNA and the words on this page) is dependent on their relative *inactivity*, or relative inertness, and this immediately disqualifies them from the agency attributed to them in accounts of this kind.

## 6. Conclusion

The modern scientific world view was built on Cartesian metaphysics and the incommensurability between cognition (mind) and physics (matter), and later life and physics (the autonomy of biology). Boltzmann's view of the second law of thermodynamics as a ‘law of disorder’ cemented the idea of ‘two opposing rivers’, the river of physics ‘flowing down’ to disorder and the river of life and mind ‘flowing up’ into contemporary science, and is a widely accepted cornerstone today. The consequence of bracketing the disciplines off in this way has been to severely limit the scope and theoretical reach of each leaving answers to the deepest questions, such as the very nature of life itself and its cognitive ability outside the theoretical reach of contemporary science. An expanded view of physics notably the addition of the fourth law of thermodynamics (LMEP) coupled with first law time-translation symmetry, and the self-referencing circularity of the relational ontology of ACK systems, provides the basis for a grand unified theory that unifies physics, life, information and cognition (mind), dissolves the dysfunctional myth of the two rivers, and greatly empowers the theoretical reach of each of the disciplines.

## Data Availability

This article has no additional data.

## Footnotes

1 E.g. ‘Inquiry into final causes is sterile,’ said Bacon, ‘and like a virgin consecrated to God produces nothing’ (*Nam causarum finalium inquisito sterilis est, et tanquam virgo Deo consecrata, nihil parit* [1]).

2 In a revisionist move most modern texts have changed Clausius' word ‘strives’ to ‘tends’ making it seem to conform to Boltzmann's statistical view of the second law, a view which Clausius did not share.

3 By stressing here ‘information’ in the meaningful sense, or ‘aboutness’ the intent is to completely disassociate the term ‘information’ from the Shannon or its informational theoretic sense which has, despite rampant misuse, nothing at all to do, as Shannon himself clearly noted, with meaningfulness (semantics).

4 In the case of more literal algorithmic computational models this boils down to the question of how do you get semantics from pure syntax?

## Appendix A. The problem with the not so truth-apt term ‘self-organizing system’

Clearly, the outcome of an argument can be turned upside down if the meaning of the terms is changed, and so pigs can fly. And so with the term ‘self-organization’ (SO),^5^ differing meanings promote erroneous conflations. SO is used today, largely for two opposing categories. The first use is for ACKs where the classic BC is an abiotic exemplar. The second is for rule-based, or programmed systems.

For example, Batten *et al*. [42] define SO as ‘a process in which the global level…emerges solely…from the rules specifying the interactions among the system's components….executed at the lower level’. But the only place where a global pattern is produced solely from rules executed in this way is in a computer, and this then begs the term ‘solely’ since the whole system is extrinsically programmed to begin with and itself sits within and is produced internally to an ACK. Chemero [43], relatedly, uses the familiar bird flocking simulation as an example of SO noting that there is ‘no bird in charge’ and that to flock each bird only needs to enact a set of ‘simple rules’. The problem is the same. SO, so defined brackets the whole environment or relational ontology out of the term, which from the point of view argued here is nearly everything. All rule-based systems (all systems that employ RICs) sit internal to ACKs which account not only for the selection of the rules, but the requisite reading, writing and translation of them to function or have a reason for doing so (see [20] for further discussion). The term ‘ACK’ or 'autocatakinetic’ system is used to make this very important distinction. Finally, making the concluding point for this appendix that where the erroneous conflation is made and this distinction is not then there is sure to be extra-physical (= ‘magic’) agential or causal smuggling taking place and the ghost in the machine remains.

## Appendix B. The fourth law (LMEP), MEP, MEPP, MaxEP and erroneous conflations

Since our identification of the fourth law or LMEP now close to three and a half decades ago there have been an increasing number of erroneous statements of it appearing in the literature which themselves then become references compounding further error. Critiquing each of these is far beyond the space allocated here (see [19,31] for more detail), but to head off further error the main kind of errors will briefly be addressed. First to nomenclature. ‘MEP’ our original acronym for the law, was unfortunately adopted a decade or so ago for a body of work on an erroneous ‘MEP’ principle (call this ‘MEP***_w_***’). Others have stated LMEP correctly but called it by another acronym used sometimes also for MEP***_w_***. At this point, following others, we put the ‘L’ into our acronym (LMEP) to make the distinction. Not long after, following Martinez-Castilla & Martinez-Khan [44] who identified LMEP as the fourth law, an idea with which we and others were already onboard with, we, and others started referring to LMEP as ‘The fourth law’. LMEP still continues by some to have other names pinned to it such as MaxEP, MEPP and others that are also used simultaneously for MEP***_w_***.

As those studied in the literature would know MEP***_w_*** is a failed hypothesis abandoned 30 to 40 years ago by the Prigogine school and others like us working on the problem of evolutionary ordering. Roughly, MEP***_w_*** wants to claim that for systems sufficiently far from equilibrium that entropy production increases to a maximum (monotonically goes up) to some imaginary steady or stationary state where it is maximized. Beyond the literature, the study of living systems, and even abiotic ACKs show readily that this principle is simply false. In simplest terms (e.g. [5,8,20]), ACKs are developmental systems, and as they grow are necessarily subject to generalized allometry, e.g. surface/volume changes, freezing out of degrees of freedom, or generalized senescence that means at some point their specific entropy production goes down. This is easily seen in the development of a single BC, and shows just why cells divide rather than just grow big (e.g. [8,20] for detailed study). Living systems are subject to all the same generics amplified even further. Further if one understands the ‘what’, ‘why’ and ‘how’ of living things (§5a) (arbitrariness with respect to local potentials) the behavioural ‘strategies’ living systems employ will mean dramatic ups and downs in their entropy production simply due to what they are and why they were physically selected in the first place [4]. The fourth law, valid in all ranges, near and far from equilibrium answers an entirely different question than the failed MEP***_w_*** and it is important that these two not be conflated. To miss this point is not only to misunderstand the physics but the whole origin, nature and evolution of life and cognition.

Confusions concerning Prigogine's minEP principle (which does not, in fact, conflict with LMEP) and Jayne's MaxEnt, an information-theoretical inference method are discussed elsewhere [20,31].
